# *Schinopsis brasiliensis* Engler—Phytochemical Properties, Biological Activities, and Ethnomedicinal Use: A Scoping Review

**DOI:** 10.3390/ph15081028

**Published:** 2022-08-20

**Authors:** Ladaha Pequeno Menna Barreto Linhares, Bruna Vanessa Nunes Pereira, Maria Karoline Gomes Dantas, Wislayne Mirelly da Silva Bezerra, Daniela de Araújo Viana-Marques, Luiza Rayanna Amorim de Lima, Pedro Henrique Sette-de-Souza

**Affiliations:** 1 Programa de Pós-Graduação em Saúde e Desenvolvimento Socioambiental, Universidade de Pernambuco–Garanhuns, Recife 55294-902, Brazil; 2 Faculdade de Odontologia, Universidade de Pernambuco–Arcoverde, Recife 56503-146, Brazil

**Keywords:** *Schinopsis brasiliensis*, phytochemistry, ethnopharmacology, antimicrobial

## Abstract

Brazil has the most incredible biodiversity globally and has a vast storehouse of molecules to be discovered. However, there are no pharmacological and phytochemical studies on most native plants. Parts of *Schinopsis brasiliensis* Engler, a tree from the *Anacardiaceae* family, are used by several traditional communities to treat injuries and health problems. The objective of this scoping review was to summarize the pharmacological information about *S. brasiliensis*, from ethnobotanical to phytochemical and biological studies. Data collection concerning the geographical distribution of *S. brasiliensis* specimens was achieved through the Reflora Virtual Herbarium. The study’s protocol was drafted using the Preferred Reporting Items for Systematic reviews and Meta-Analyses extension for Scoping Reviews (PRISMA-ScR). The search strategy used the keyword “*Schinopsis brasiliensis*” in the databases: PUBMED, EMBASE, SCOPUS, Science Direct, Web of Science, SciFinder, and SciELO. Rayyan was used for the selection of eligible studies. In total, 35 studies were included in the paper. The most recurrent therapeutic indications were for general pain, flu and inflammation. The bark was the most studied part of the plant. The most used preparation method was decoction and infusion, followed by syrup. Phytochemical investigations indicate the presence of tannins, flavonoids, phenols, and polyphenols. Most of the substances were found in the plant’s leaf and bark. Important biological activities were reported, such as antimicrobial, antioxidant, and anti-inflammatory. *S. brasiliensis* is used mainly by communities in the semi-arid region of northeastern Brazil to treat several diseases. Pharmacological and phytochemical studies together provide scientific support for the popular knowledge of the medicinal use of *S. brasiliensis*. In vitro and in vivo analyses reported antimicrobial, antioxidant, anti-inflammatory, antinociceptive, cytotoxic, photoprotective, preservative, molluscicidal, larvicidal, and pupicidal effects. It is essential to highlight the need for future studies that elucidate the mechanisms of action of these phytocompounds.

## 1. Introduction

Medicinal plants have been used in many cultures for thousands of years, and information on the use of natural resources plays a vital role in discovering new products from plants as therapeutic agents [1]. Brazil is the country with the most extensive biodiversity globally, being a potential storehouse of molecules still not discovered, envisioning their use as a source of therapeutic resources. However, there are still no pharmacological and phytochemical studies on most native plants [2].

**Figure 1:**
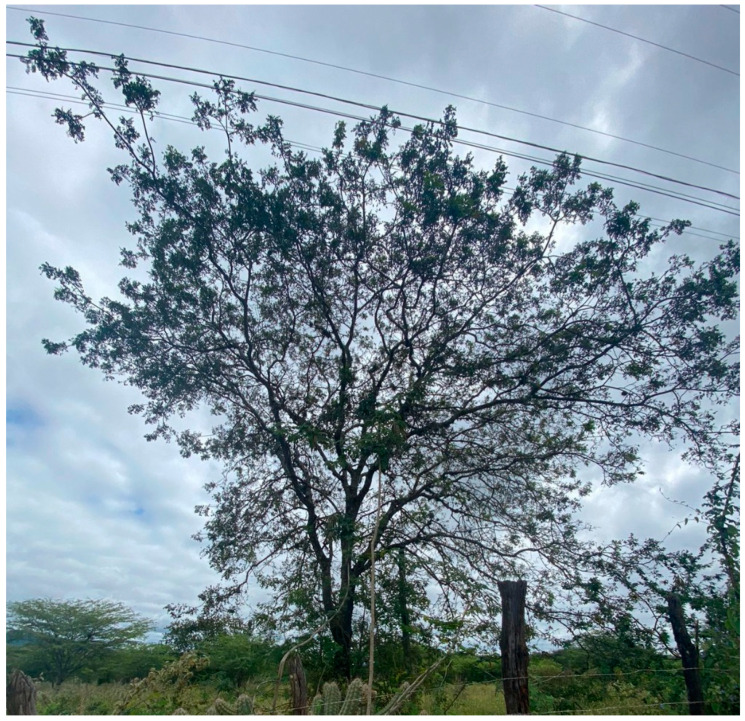
*Schinopsis brasiliensis* Engl. Image captured by the authors (Arcoverde/Pernambuco/Brazil—July/2022).

*Schinopsis brasiliensis* Engler is a tree of the Anacardiaceae family, of deciduous behavior, and can reach a height of 20 m (Figure 1) [3]. Its bark is gray, almost black, rough, and detaches in irregularly square portions, up to 30 mm thick [4]. *S. brasiliensis* is a native tree of the Caatinga, a unique Brazilian Biome located in the semiarid region of Brazilian northeastern, found from latitude 5° S in Ceará and Rio Grande do Norte, to 20° S in Mato Grosso and Minas Gerais [4,5].

It is popularly known in Brazil as “braúna”, “baraúna”, “braúna-do-sertão”, “braúna-parda”, “quebracho”, “chamacoco” and “chamucoco” [6,7] and in Bolivia as “soto” [3]. *S. brasiliensis* is used for medicinal purposes by several communities, depending on the location studied [8]. According to ethnobotanical surveys, several parts of *S. brasiliensis* are used for the treatment of various injuries and diseases, such as inflammatory disorders [9,10,11], diarrhea [9,12,13], influenza [9,13,14,15,16,17], cough [12,13,15], and sexual impotence [9,10,13]. The species has already proven biological activities, such as antinociceptive [18,19], anti-inflammatory [18,19], antioxidant [18,19,20,21,22] antimicrobial [23,24,25,26,27], and photoprotective [27,28].

Phytochemical investigations indicate the presence of tannins [10,22,27,29,30,31,32], flavonoids [27,30,31,32,33], phenols [10,27], saponins [29,33], triterpenes [29,33], quinones [10], alkaloids [29], polyphenols [31], gallic acid [31], condensed tannins, and phenolic acid [33].

Although some research reports the chemical composition and pharmacological activities of *S. brasiliensis* extracts, no review has been published to critically summarize these studies and suggest the use of the plant as a source of molecules of interest for future applications. Thus, the objective of this scoping review was to synthesize pharmacological information about *S. brasiliensis*, from ethnobotanical to phytochemical and biological studies.

## 2. Material and Methods

### 2.1. Geographical Distribution of S. brasiliensis

The collection of data concerning the geographical distribution of identified *S. brasiliensis* specimens was achieved through the Reflora Virtual Herbarium (Reflora Program—CNPq-https://reflora.jbrj.gov.br/reflora/herbarioVirtual, accessed on 28 May 2021). The previous authorization was conceded, and latitude and longitude data of each collected specimen were retrieved. Then, we plotted a map using RStudio 1.4 (through ‘geobr’ and ‘ggspatial’ packages) with the retrieved geographical data.

### 2.2. Protocol and Registration

The study’s protocol was drafted using the Preferred Reporting Items for Systematic reviews and Meta-Analyses extension for Scoping Reviews (PRISMA-ScR) [34]. The final protocol was registered with the Open Science Framework on 4 June 2021 (https://doi.org/10.17605/osf.io/drjns, accessed on 4 June 2021).

### 2.3. Eligibility Criteria

Studies were included if: (i) published until 25 May 2021; (ii) a peer-reviewed publication; (iii) written in English, Portuguese, or Spanish; (iv) that had described the use of *Schinopsis brasiliensis*. Non-original articles were excluded, such as monographs, dissertations, theses, bibliographic reviews, letters, comments, editorials, or book chapters and studies that did not describe an antimicrobial, ethnobotanical, or a phytochemical approach to *S. brasiliensis*.

### 2.4. Search Strategy and Information Sources

The search strategy used the keyword “*Schinopsis brasiliensis*” in the following bibliographic databases: PUBMED, EMBASE, SCOPUS, Science Direct, Web of Science, SciFinder, and SciELO. The final search results of each database were exported and downloaded in CIW or RIS format. The files were imported into the online platform of Rayyan QCRI (RRID:SCR_017584-PMID: 27919275-https://www.rayyan.ai, accessed on 4 June 2021), and duplicates were removed.

### 2.5. Selection of Sources of Evidence

Rayyan was used to select eligible studies [35]. Based on the eligibility criteria, two reviewers (MKGD and WMSB) independently evaluated the same titles, abstracts, and full text of all publications identified by the searches. The disagreements on study selection and data extraction were resolved by consensus and discussion with a third reviewer (PHSS), when needed. The intra- and interobserver Kappa coefficients were performed using 70% of previously identified studies. The selection of sources was carried out until 25 May 2021. However, a new search was performed on 5 July 2022, to update the selected studies.

### 2.6. Data Items and Synthesis of Results

The data of selected studies according to the study approach (ethnobotanical, antimicrobial, phytochemical) were extracted and summarized as shown in the Tables. Study localization, plant part, extraction product, the method for extraction, compound class, identified compound, biological activity, and therapeutic indication were collected for each study.

## 3. Results

### 3.1. Geographical Distribution of S. brasiliensis

**Figure 2:**
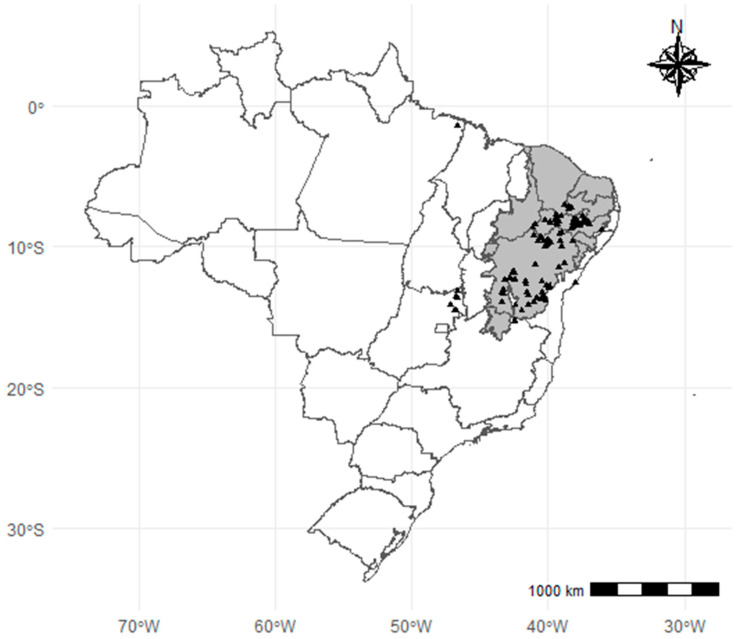
Geographical distribution of identified *Schinopsis brasiliensis* Engl specimens from the Reflora Virtual Herbarium collection found in Brazil. (Map plotted using RStudio 1.4 with ‘geobr’ and ‘ggspatial’ packages).

Based on the Reflora Virtual Herbarium data, we observed that the Caatinga Biome (northeastern Brazil) contained the majority of identified *Schinopsis brasiliensis* Engl specimens (Figure 2). Five specimens were identified in other regions, one in northeastern Pará and four in northeastern Goiás. There is a large concentration of specimens identified between 7° S/15° S and 36° W/43° W.

### 3.2. Summary of the Articles

**Figure 3:**
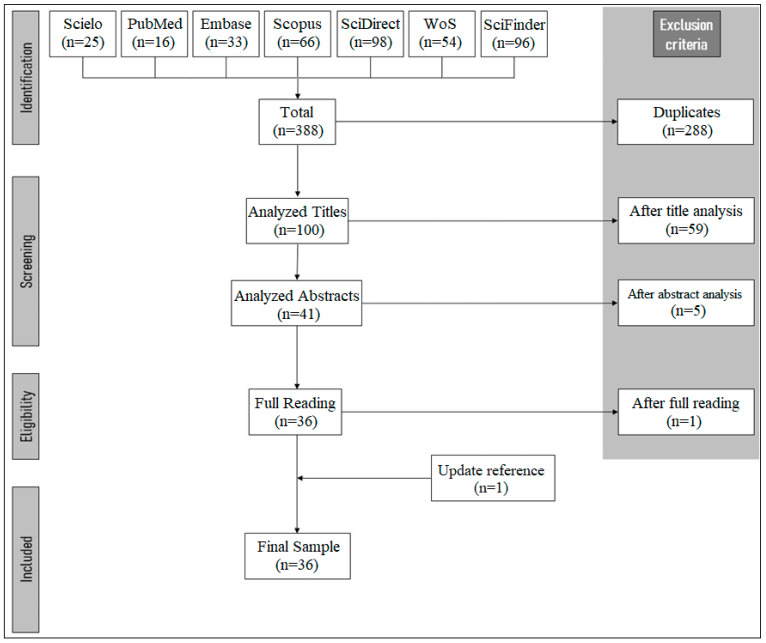
Flow chart of the articles selection process according to PRISMA-ScR.

A total of 388 titles were retrieved using the search strategy. After the removal of duplicates, 100 unique studies were independently evaluated by reviewers using eligibility criteria (Figure 3). The intra-observer Kappa coefficient was 0.96 (C.I. 0.76–1.00) and the inter-observer was 0.92 (C.I. 0.62–1.00). After full reading and updating references, 36 published studies were included in this scoping review.

### 3.3. Ethnobotanical Studies

Ethnobotanical studies have shown different ways to use *S. brasiliensis* by local communities, besides its uses for treating various symptoms (Table 1).

**Figure 4:**
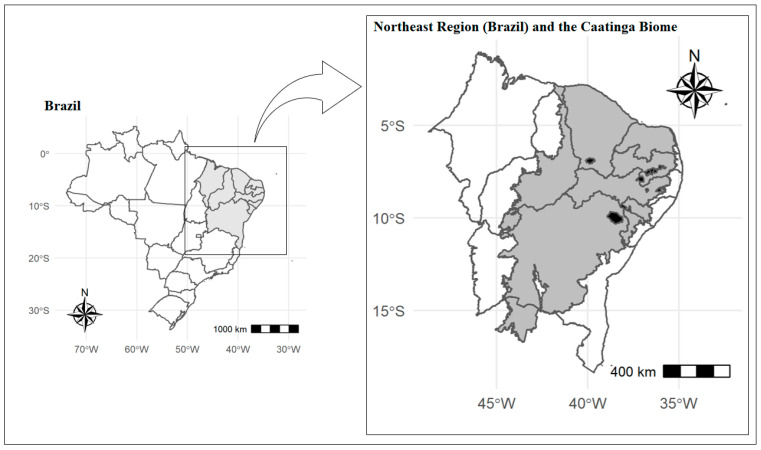
Regions of the Ethnobotanical Surveys (black) conducted in Brazil, with emphasis on the Caatinga Biome (gray).

All ethnobotanical studies presented are Brazilian (*n* = 11,100%), from the Northeast region (Figure 4). General pain (tooth, ear, throat, stomach, liver, back, nerves, and menstrual cramps) was the most recurrent therapeutic indication (*n* = 8; 72.72%), followed by influenza (*n* = 6; 54.54%), and inflammation (*n* = 3; 27.27%). The barks were the most studied part of the plant (*n* = 7, 63.63%). The most used preparation method was the tea-decoction or infusion (*n* = 7, 63.63%). Thus, we observed the way that *S. brasiliensis* is used as a medicinal drug and the preparation mode. 

### 3.4. Phytochemistry Studies

Eleven studies showed the phytochemical classes of *S. brasiliensis*, without identifying the compounds (Table 2). We noted that the plant is a phenolic compound source. Tannins are identified almost always (*n* = 10; 90.9%), although flavonoids (*n* = 7; 63.63%), phenols and polyphenols (*n* = 3; 27.27%), triterpenes and saponins (*n* = 2; 18.18%) are also observed in the papers. A lot of studies had isolated many phytocompounds from *S. brasiliensis*, according to the plant’s part (Table 3).

Eight studies described 64 isolated chemical compounds from *S. brasiliensis*. Polyphenols were the most prevalent chemical group identified (*n* = 15; 23.43%), followed by terpenes (*n* = 13; 20.31%). Most of the compounds were found in the leaves (*n* = 31; 48.43%).

### 3.5. Antimicrobial Activity

Fourteen studies presented results on the antibacterial activity of *S. brasiliensis* extracts against 17 bacteria, eight Gram-negative and nine Gram-positive. Table 4 summarizes the studies that reported the antibacterial activity of *S. brasiliensis* extracts. Notably, the leaf extract of *S. brasiliensis* showed antifungal activity against *C. albicans*, *C. tropicalis*, and *C. krusei* [6,22]. In addition, Formiga-Filho et al. [26] noted that the association of *S. brasiliensis* bark extract with low-power laser increases its activity against *E. coli*, *S. aureus*, *P. aeruginosa*, and *E. faecalis*.

In these studies, the bark was the most used plant structure (*n* = 7; 50%), followed by the leaves (*n* = 6; 44.8%). The ethanolic extract was used in 44.8% of the studies (*n* = 6). The most cited bacterium in the studies was *Staphylococcus* spp. (*n* = 9; 63.5%). The range of Minimum Inhibitory Concentration (MIC) varied as to concentrations, being 1 µL/µL for *E. faecalis* [1], 0.23 µg/mL for *Escherichia coli* [43], 0.004 µL/µL for *P. aeruginosa* [1] and 10 µg/mL for *K. pneumoniae* [43].

Besides the antimicrobial activity of the extracts, two studies evaluated the antibacterial effect of controlled release systems containing *S. brasiliensis*. The production of chitosan microparticles-loaded *S. brasiliensis* bark extract would be an alternative for the use of the extract in dentistry due to the improved organoleptic properties [23]. The MIC values of these microparticles were lower than that observed for the hydroalcoholic extract (0.25 mg/mL and 0.50 mg/mL, respectively). Furthermore, the microparticles inhibited biofilm development and growth of *E. faecalis* in 24 h. Through cytotoxicity analyses performed by Sette-de-Souza et al. [23], it was proven that microparticles are safe for use in the treatment of *Enterococci* infections and in dentistry due to their potential to inhibit biofilm development.

Oliveira et al. [43] showed that *S. brasiliensis* nanoparticles associated with ceftriaxone showed inhibitory activity against *E. coli*, including against ceftriaxone-resistant strains. These results express the capacity and importance of the use of controlled-release systems in the delivery of atypical pharmaceutical ingredients, demonstrating to be an excellent possibility for the treatment of infections caused by multidrug-resistant bacteria.

### 3.6. Antioxidant Activity 

The antioxidant activity of *S. brasiliensis* extracts was evaluated in six studies (Table 5), through four tests: Oxygen Radical Absorbance Capacity-ORAC [20], 2,2-Diphenyl-1-Picryl-Hydrazyl-DPPH [19,20,22,27,28], β-Carotene [19,27] and Trolox Equivalent Antioxidant Capacity-TEAC [21]. Twenty-three results were obtained from the six studies. The DPPH (*n* = 11; 47.82%) and β-carotene (*n* = 9; 39.13%) methods were most used.

### 3.7. Cytotoxic Activity

The cytotoxic activity was evaluated in different experimental models (Table 6). The bark was the most used part of *S. brasiliensis* (*n* = 13; 52%). In vivo studies (*n* = 10; 40%) used model *Artemia salina* (*n* = 9; 90%) [1,22,45,46,47] and *Ceriodaphnia dubia* (*n* = 1; 10%) [47] were tested and the LC_50_ ranged from 1.91 mg/mL to 962.97 µg/mL. In vitro studies (*n* = 15; 60%) evaluated cytotoxicity against fibroblasts cell lines (*n* = 3; 20%) [39,47] or cancer lines (*n* = 12; 80%) [39]. In this way, *S. brasiliensis* should be a promising anticancer agent.

### 3.8. Other Biological Activities

Other biological activities of *S. brasiliensis* extracts have also been reported, such as photoprotective against Ultraviolet B radiation [27,28], sunscreen preservative [48], molluscicidal [46], larvicidal [45,46,47], pupicidal [45,47], ovicidal [45,47], anti-inflammatory [18,19], nociceptive [18,19], antihemolytic [23,24,27] and enzyme inhibiting [47] (Table 7).

A sun Protection Factor of 6 UVB was observed for the ethanolic extract of the bark of *S. brasiliensis* [27]. The bark extract of the plant can also be used in photoprotective formulations since it has preservative aspects, according to the analytical methods used [48].

Molluscicidal and larvicidal activities were observed in the study with *S. brasiliensis* bark. Through the method using *Biomphalaria glabrata*, it was possible to observe that the chloroform fraction of the ethanolic extract resulted in an LC_90_ of 68 μg/mL, and an ethyl acetate fraction of 73 μg/mL [46]. The larvicidal activity was also observed against *Aedes aegypti* larvae using the method recommended by the World Health Organization (WHO) for the ethyl acetate (LC_50_: 345 μg/mL), hexane (LC_50_: 527 μg/mL), and chloroform (LC_50_: 583 μg/mL) fractions [46]; while the ethanolic extract of the seeds was able to eliminate *A. aegypti* larvae (field-collected larvae-LC_50_: 580.9 µg/mL; insecticide-susceptible larvae-LC_50_: 661.6 µg/mL) [45]. The pupicidal potential of the ethanolic extract of the seeds was also evaluated, being described as an excellent activity, both for pupae collected in the field of *A. aegypti* (LC_50_: 32.9 µg/mL), and for those susceptible to insecticide (LC_50_: 40.6 µg/mL) [45]. In another study, Barbosa et al. [47] studied the larvicidal activity of the crude extract of *S. brasiliensis* seeds, using the Konishi et al. (2008) adapted and WHO (2005) adapted methods. The authors observed 100% death against L1 and L4 *Aedes aegypti* larvae, obtained in 24 h, LC_50_ of 6.01 mg/mL and 6.14 mg/mL and in 48 h LC_50_ of 5 mg/mL and 1 mg/mL, respectively.

The nociceptive activity was verified by formalin-induced licking behavior and/or through paw edema [18,19]. The hydroethanolic extract of *S. brasiliensis* bark and its ethyl acetate fraction reduced the licking time of mice by 40% when applied 30 mg/kg [18].

The anti-hemolytic activity was observed in three studies. The ethanolic extracts of the bark (*n* = 2; 66.66%) obtained the following results: 43.83% [27] inhibition of erythrocyte hemolysis, while the other one showed the IC50 (maximum concentration to obtain 50% inhibition) 50.27 mg/mL [24] as a result. The hydroalcoholic extract of the barks (*n* = 1; 33.33%) resulted in IC50 92.66 mg/mL [23].

## 4. Discussion

This review reports on the geographical distribution, ethnopharmacological use, biological activities, toxicology, and pharmacology of *Schinopsis brasiliensis*. This plant treats some health problems, mainly in the Caatinga population. The results of the ethnobotanical surveys show variability in the use of parts of the plant to treat several diseases. The difference in indications of use can be explained by the diversity of bioactive molecules found in *S. brasiliensis*, considering that the environmental conditions, such as temperature, soil, and humidity, directly impact the chemical composition of the plants.

This work observed that most specimens of *S. brasiliensis* identified in Brazil were from the Caatinga Biome. However, the species is reported to be found in the Chaco (Bolivia and Paraguay) and the Brazilian Cerrado, up to near latitude 20° S. Despite this finding, there is no specific information regarding the population density of *S. brasiliensis* in this region [3].

This location of *S. brasiliensis* may explain the concentration of studies in the Caatinga Biome, a large natural region, being the only exclusively Brazilian biome [49]. It has only two most expressive climates: the rainy period and the dry period [38]. These environmental stress factors can directly interfere with producing the plant’s secondary metabolites [50], resulting in several applications.

The great diversity of phytocompounds present in *S. brasiliensis* may be related to the indications of popular use. The phytochemical characterization of *S. brasiliensis* reveals numerous bioactive molecules belonging to several metabolic classes with reported biological activities. Secondary metabolites act by retarding and/or inhibiting the action of free radicals. The observed antioxidant capacity is probably due to the high content of compounds, such as flavonoids, tannins, and phenolic acids. These compounds could donate electrons, thus stabilizing free electrons, in addition to inactivating superoxide anions and peroxide radicals [51].

Tannins have astringent properties, precipitating proteins, and being favorable for antibacterial and antifungal effects [52]. Once administered via the oral route, they promote antidiarrheal and antiseptic effects. Due to the tannin-protein/polysaccharides complex, formed in the precipitation of proteins, creating a protective layer [52], they may exert a healing effect [53]. Thus, the presence of tannins [10,23,24,27,28,29,30,33,38], such as corilagin [39], in *S. brasiliensis* may explain the use of the plant to treat diarrhea [9,12,13], stomach pain [37], verminosis [36], infection [11], and fracture [13]. Phenolic compounds are related to antioxidant activities, pharmacological activities, modulation of different enzymes, interactions with receptors, and cell cycle regulations [54].

Flavonoids are compounds that can inhibit or retard enzymatic actions, characterizing their antioxidant action [55]. Their anti-inflammatory potential is associated with the inhibition of enzymes [56] such as cyclooxygenase (COX), lipoxygenase [57], and the inhibition of COX-2 and nitric oxide synthase [58]. Recently, the affinity between some *S. brasiliensis* phytocompounds and COX-1, COX-2, and LOX were evaluated, showing a promising anti-inflammatory activity [19]. Thus, flavonoids may have anti-inflammatory, antioxidant, antiallergic, antiviral, antithrombotic, and anticarcinogenic actions [55,59]. Catechins and derivatives found in *S. brasiliensis* extracts may be related to these described activities. Thus, this explains why in folk medicine *S. brasiliensis* is used to treat diseases of the respiratory tract [9,12,14,15,16,17], earache [36], toothache [36], inflammation [9,10,11], menstrual cramps [11], and fractures [9,13,16].

Because analgesic and anti-inflammatory drugs have significant adverse effects, new prototype drugs are of great interest to the scientific community. Terpenes are secondary metabolites, best known for their action on the Central Nervous System (sedative, tranquilizing, anticonvulsant, anxiolytic, and nociceptive effects). These pharmacological activities are similar to opioids [60,61,62]. In addition, terpenes are good antimicrobial agents through their ability to permeabilize and depolarize the cytoplasmic membranes of microorganisms. *S. brasiliensis* is rich in terpenes, such as myrcene, α-pinene and linalool. Therefore, one can associate the activity of terpenes with the use for sore throat [9], earache [36], toothache [36], pain in the nerves and spine [17], pain in the stomach and liver [37], reported in ethnobotanical surveys. In addition, terpenes can be attributed to nociceptive activity in rats [18,19].

Saponins are related to the defense mechanism of plants, being found in tissues that are more susceptible to attacks by fungi, insects, and bacteria [63]. They can alter membrane permeability related to ichthyotoxic and molluscicidal activities [64]. The literature reports their use as expectorants and diuretics [64] and their ingestion for stool hardening without affecting intestinal motility [65]. Thus, the saponins present in *S. brasiliensis* may justify its popular use for coughs [12,13,15], influenza [9,13,14,15,16,17,66], cold [9,14], diarrhea [9,12]. Moreover, this class of phytocompounds can justify the results found against *Biomphalaria glabrata* [46] and *Aedes aegypti* [45,46,47].

The replacement of synthetic insecticides has become a necessity, mainly related to pest resistance to these products. Besides this issue, to control populations of disease vectors such as mosquitoes, for example, larvicidal and pupicidal activities are necessary. Another critical situation is that some mollusks can be part of the biological cycle of helminths—hence the need to control these animals. 

The importance of the species and its use for therapeutic purposes is observed since these phytochemical compounds presented have different biological activities.

## 5. Conclusions and Perspectives

We noticed that *S. brasiliensis* is used mainly by communities in the Northeast of Brazil, especially in the Caatinga, to treat various diseases. The traditional use of *S. brasiliensis* varies according to the part and the community studied. However, the difference in these reports can be attributed to the richness of bioactive compounds present in the plant.

On the other hand, the pharmacodynamic and pharmacokinetic properties of *S. brasiliensis* extracts have not been determined. Thus, future investigations are necessary to determine these parameters to understand the bioavailability of the phytocompounds from *S. brasiliensis*. Finally, it is essential to highlight the need for future studies to explore and elucidate the mechanisms of action of these phytocompounds.

## Figures and Tables

**Table 1:** List of therapeutic indications of *Schinopsis brasiliensis* Engler according to the results of the ethnobotanical surveys.

Therapeutic Indication	Location	Used Part	Preparation	Reference
Antitussive, diarrhea, and dysentery	Cabaceiras/PB, São João do Cariri/PB, Serra Branca/PB, Monteiro/PB	Bark	Decoction, syrup	Agra et al. [12]
Cold and flu	Alagoinha/PE	Bark	Infusion, Syrup	Albuquerque [14]
Antitussive and flu	Alagoinha/PE	Bark	Decoction, Syrup	Albuquerque and Andrade [15]
Fracture, Inflammation, Sexual Impotence, Sore Throat Cold, Flu, and Diarrhea	Unreported	Bark, Leaf, Fruit, Seed, Resin	Unreported	Albuquerque et al. [9]
Antihisteric, nervosthenic, tonic, toothache, earache, verminosis	Campina Grande/PB	Resin, Bark	Tincture, Decoction, Infusion	Albuquerque et al. [36]
Inflammation and Sexual Impotence	Piranhas/AL, Delmiro Gouveia/AL	Bark	Unreported	Almeida et al. [10]
Menstrual Cramps, Inflammation, Infection	Altinho/PE	N/E	Unreported	Ferreira-Júnior et al. [11]
Prostate, anticoagulant, flu, and bones	Jeremoabo/BA	Bark	Maceration, Tea, Syrup	Gomes and Bandeira [16]
Back pain, nerve pain, flu	Monteiro/PB	Flower	Decoction	Pereira-Júnior et al. [17]
Stomach pain, liver pain	Assaré/CE	Leaf	Decoction	Ribeiro et al. [37]
Cough, flu, diarrhea, fractures, sexual impotence	Unreported	Bark	Unreported	Silva et al. [13]

PB: Paraíba; PE: Pernambuco; AL: Alagoas; BA: Bahia; CE: Ceará.

**Table 2:** Phytochemical compounds found in *Schinopsis brasiliensis*.

Used Part	Extract	Compound	Amount	Reference
Unreported	Ethanolic	Alkaloids	-	Almeida et al. [29]
Bark	Ethanolic	Flavonoids	132.4 ± 1.76 mg/g (RE)	Lima-Saraiva et al. [27]
Bark	Ethanolic	Flavonoids	6.94 mg/g	Sette-de-Souza et al. [24]
Bark	Hydroalcoholic	Flavonoids	1.44%	Fernandes et al. [31]
Bark	Hydroalcoholic	Flavonoids	10.16 ± 0.54 mg/g	Sette-de-Souza et al. [23]
Bark	Methanolic	Flavonoids	2.63%	Araújo et al. [30]
Bark	Methanolic	Flavonoids	-	Saraiva et al. [33]
Flowers	Methanolic	Flavonoids	-	Saraiva et al. [33]
Fruit	Methanolic	Flavonoids	-	Saraiva et al. [33]
Leaves	Methanolic	Flavonoids	-	Saraiva et al. [33]
Root	Methanolic	Flavonoids	-	Saraiva et al. [33]
Seeds	Methanolic	Flavonoids	-	Saraiva et al. [33]
Bark	Unreported	Flavonoids	2.55%	Siqueira et al. [32]
Bark	Hydroalcoholic	Gallic acid	-	Fernandes et al. [31]
Heartwood	Butanol	Phenol	501.94 ± 10.49 mg/g (GAE)	Moreira et al. [19]
Root Bark	Butanol	Phenol	505.25 ± 11.65 mg/g (GAE)	Moreira et al. [19]
Heartwood	Chloroform	Phenol	474.38 ± 7.07 mg/g (GAE)	Moreira et al. [19]
Root Bark	Chloroform	Phenol	525.31 ± 2.67 mg/g (GAE)	Moreira et al. [19]
Bark	Ethanolic	Phenol	-	Almeida et al. [10]
Bark	Ethanolic	Phenol	493.88 ± 13.23 mg/g (TAE)	Almeida-Andrade et al. [28]
Bark	Ethanolic	Phenol	624.6 ± 0.42 mg/g (GAE)	Lima-Saraiva et al. [27]
Heartwood	Ethyl Acetate	Phenol	816.37 ± 15.40 mg/g (GAE)	Moreira et al. [19]
Root Bark	Ethyl Acetate	Phenol	648.26 ± 6.01 mg/g (GAE)	Moreira et al. [19]
Heartwood	Hexane	Phenol	19.14 ± 2.67 mg/g (GAE)	Moreira et al. [19]
Root Bark	Hexane	Phenol	76.61 ± 6.7 mg/g (GAE)	Moreira et al. [19]
Bark	Methanolic	Phenolic acid	-	Saraiva et al. [33]
Flowers	Methanolic	Phenolic acid	-	Saraiva et al. [33]
Fruit	Methanolic	Phenolic acid	-	Saraiva et al. [33]
Leaves	Methanolic	Phenolic acid	-	Saraiva et al. [33]
Root	Methanolic	Phenolic acid	-	Saraiva et al. [33]
Seeds	Methanolic	Phenolic acid	-	Saraiva et al. [33]
Bark	Ethanolic	Polyphenols	598.55 mg/g	Sette-de-Souza et al. [24]
Bark	Hydroalcoholic	Polyphenols	15.08%	Fernandes et al. [31]
Bark	Hydroalcoholic	Polyphenols	586.13 ± 9.38 mg/g	Sette-de-Souza et al. [23]
Bark	Ethanolic	Quinones	-	Almeida et al. [10]
Unreported	Ethanolic	Saponins	-	Almeida et al. [29]
Bark	Methanolic	Saponins	-	Saraiva et al. [33]
Flowers	Methanolic	Saponins	-	Saraiva et al. [33]
Fruit	Methanolic	Saponins	-	Saraiva et al. [33]
Leaves	Methanolic	Saponins	-	Saraiva et al. [33]
Root	Methanolic	Saponins	-	Saraiva et al. [33]
Seeds	Methanolic	Saponins	-	Saraiva et al. [33]
Bark	Ethanolic	Tannins	-	Almeida et al. [10]
Bark	Ethanolic	Tannins	367.12 ± 21.35 mg/g (TAE)	Almeida-Andrade et al. [28]
Bark	Ethanolic	Tannins	255.8 ± 2.06 mg/g (TAE)	Lima-Saraiva et al. [27]
Bark	Ethanolic	Tannins	15.83 mg/g	Sette-de-Souza et al. [24]
Unreported	Ethanolic	Tannins	-	Almeida et al. [29]
Bark	Hydroalcoholic	Tannins	27.12 ± 0.61 mg/g	Sette-de-Souza et al. [23]
Bark	Methanolic	Tannins	50.24%	Araújo et al. [30]
Bark	Methanolic	Tannins	-	Saraiva et al. [33]
Flowers	Methanolic	Tannins	-	Saraiva et al. [33]
Fruit	Methanolic	Tannins	-	Saraiva et al. [33]
Leaves	Methanolic	Tannins	-	Saraiva et al. [33]
Root	Methanolic	Tannins	-	Saraiva et al. [33]
Seeds	Methanolic	Tannins	-	Saraiva et al. [33]
Bark	Unreported	Tannins	5.53%	Siqueira et al. [32]
Leaves and Bark	Unreported	Tannins	78.9 ± 12.2 mg/g	Oliveira et al. [38]
Bark	Ethanolic	Triterpene	-	Almeida et al. [10]
Bark	Methanolic	Triterpene	-	Saraiva et al. [33]
Flowers	Methanolic	Triterpene	-	Saraiva et al. [33]
Fruit	Methanolic	Triterpene	-	Saraiva et al. [33]
Leaves	Methanolic	Triterpene	-	Saraiva et al. [33]
Root	Methanolic	Triterpene	-	Saraiva et al. [33]
Seeds	Methanolic	Triterpene	-	Saraiva et al. [33]

TAE: Tannic acid equivalent; GAE: Gallic acid equivalents; RE: Rutin equivalent.

**Table 3:** Isolated compounds from *Schinopsis brasiliensis*.

Isolated Compound	Class	Plant Part	Reference
Sylvestrene	Alkene	Leaves	Donati et al. [20]
Quercetin- O- (O- galloyl) –hexoside	Benzoate	Leaves	Reis-Luz et al. [39]
Methyl 6-eicosanyl-2-hydroxy-4-methoxybenzoate	Benzoate	Bark	Cardoso et al. [40]
Urundeuvin A	Benzopyran	Branch	Reis-Luz et al. [39]
Chlorogenic acid	Carboxylic acid	Bark	Reis-Luz et al. [39]
Citric Acid	Carboxylic acid	Bark	Reis-Luz et al. [39]
Digalloyl Quinic Acid	Carboxylic acid	Bark	Reis-Luz et al. [39]
Quinic acid	Carboxylic acid	Bark	Reis-Luz et al. [39]
Chlorogenic acid	Carboxylic acid	Branch	Reis-Luz et al. [39]
Quinic acid	Carboxylic acid	Branch	Reis-Luz et al. [39]
Quinic acid	Carboxylic acid	Leaves	Reis-Luz et al. [39]
Cajobin	Chalcone	Root bark	Moreira et al. [19]
Luxenchalcone	Chalcone	Root bark	Moreira et al. [19]
5α, 8α-epidioxyergosta-6,22-dien-3-b-ol	Cholestane	Bark	Cardoso et al. [40]
4,2′,4′-tri-hydroxichalcona-(3→O→4″)-2‴,4‴,-dihydroxiccalcona	Flavonoid	Bark	Cardoso et al. [41]
Apigenin	Flavonoid	Bark	Lima-Saraiva et al. [27]
Catechin	Flavonoid	Bark	Lima-Saraiva et al. [27]
Epicatechin	Flavonoid	Bark	Lima-Saraiva et al. [27]
Ethyl-*O*-β-D-(6′-*O*-galloyl)-glucopyranoside	Flavonoid	Branch	Reis-Luz et al. [39]
Catechin	Flavonoid	Fruit	Saraiva et al. [33]
(2R *, 3R *, 2″R *, 3″R *)-7-hydroxy-4′-methoxy-flavanone-(3→3″)-3‴, 7″-di-hydroxy-4‴-methoxyflavone	Flavonoid	Leaves	Cardoso et al. [41]
4,2′,4′-tri-hydroxichalcona-(3→O→4″)-2‴,4‴,-dihydroxiccalcona	Flavonoid	Leaves	Cardoso et al. [41]
Myricitrin *O*-gallate	Flavonoid	Leaves	Reis-Luz et al. [39]
Quercetin gallopentosis	Flavonoid	Leaves	Reis-Luz et al. [39]
Quercetin- O- hexosíde	Flavonoid	Leaves	Reis-Luz et al. [39]
Gallic acid	Gallate	Bark	Fernandes et al. [31]
Gallic acid	Gallate	Bark	Lima-Saraiva et al. [27]
Gallic acid	Gallate	Heartwood	Moreira et al. [19]
Gallic acid	Gallate	Leaves	Fernandes et al. [31]
Gallic acid	Gallate	Leaves	Lima-Saraiva et al. [27]
Gallic acid	Gallate	Root	Lima-Saraiva et al. [27]
Penta-*O*-galloyl-β-D	Gallotannin	Bark	Reis-Luz et al. [39]
*O*-galloylnorbergenin	Gallotannin	Branch	Reis-Luz et al. [39]
Penta-*O*-galloyl-β-D	Gallotannin	Branch	Reis-Luz et al. [39]
Penta-*O*-galloyl-β-D	Gallotannin	Leaves	Reis-Luz et al. [39]
C_20_H_28_O_23_	Not identified	Bark	Reis-Luz et al. [39]
C_30_H_20_O_9_	Not identified	Bark	Reis-Luz et al. [39]
C_31_H_24_O_14_	Not identified	Bark	Reis-Luz et al. [39]
C_46_H_36_O_21_	Not identified	Bark	Reis-Luz et al. [39]
C_28_H_24_O_17_	Not identified	Branch	Reis-Luz et al. [39]
C_45_H_24_O_14_	Not identified	Branch	Reis-Luz et al. [39]
C_14_H_8_O	Not identified	Leaves	Reis-Luz et al. [39]
C_18_H_26_O_14_	Not identified	Leaves	Reis-Luz et al. [39]
C_26_H_36_O_11_	Not identified	Leaves	Reis-Luz et al. [39]
C_28_H_24_O_17_	Not identified	Leaves	Reis-Luz et al. [39]
C_30_H_22_O_9_	Not identified	Root bark	Moreira et al. [19]
C_46_H_36_O_12_	Not identified	Root bark	Moreira et al. [19]
Methyl Gallate	Phenol Compound	Root bark	Moreira et al. [19]
Cynamic Derivate	Phenolic acid	Bark	Saraiva et al. [33]
Cynamic Derivate	Phenolic acid	Flowers	Saraiva et al. [33]
Cynamic Derivate	Phenolic acid	Fruit	Saraiva et al. [33]
Cynamic Derivate	Phenolic acid	Leaves	Saraiva et al. [33]
Cynamic Derivate	Phenolic acid	Root	Saraiva et al. [33]
Cynamic Derivate	Phenolic acid	Seeds	Saraiva et al. [33]
Estragole (4-allylanisole)	Phenols	Leaves	Donati et al. [20]
Daucosterol	Phytosterol	Heartwood	Moreira et al. [19]
2-hydroxy-4-methoxyphenol-1-*O*-β-D-(6′-*O*-galloyl)-glucopyranoside	Polyphenol	Bark	Reis-Luz et al. [39]
Galloyl quinic acid	Polyphenol	Bark	Reis-Luz et al. [39]
Proanthocyanidin	Polyphenol	Bark	Saraiva et al. [33]
2-hydroxy-4-methoxyphenol-1-*O*-β-D-(6′-*O*-galloyl)-glucopyranoside	Polyphenol	Branch	Reis-Luz et al. [39]
Di-*O*-galloyl-2,3-(S)-hexahydroxydiphenoy1-scyllo-quercitol	Polyphenol	Branch	Reis-Luz et al. [39]
Galloyl quinic acid	Polyphenol	Branch	Reis-Luz et al. [39]
Hexagalloyl-hexoside	Polyphenol	Branch	Reis-Luz et al. [39]
Proanthocyanidin	Polyphenol	Fruit	Saraiva et al. [33]
Digallic acid	Polyphenol	Leaves	Reis-Luz et al. [39]
Ethyl 2,4-dihydroxy-3-(3,4,5-trihydroxybenzoyl)oxybezoate	Polyphenol	Leaves	Reis-Luz et al. [39]
Hexagalloyl-hexoside	Polyphenol	Leaves	Reis-Luz et al. [39]
Tetra-*O*-galloyl-glucose	Polyphenol	Leaves	Reis-Luz et al. [39]
Proanthocyanidin	Polyphenol	Root	Saraiva et al. [33]
Ellagic Acid	Polyphenol	Root bark	Moreira et al. [19]
Corilagin	Tannin	Branch	Reis-Luz et al. [39]
Aromadendrene	Terpene	Leaves	Donati et al. [20]
Eucalyptol (cineol)	Terpene	Leaves	Donati et al. [20]
Globulol	Terpene	Leaves	Donati et al. [20]
Guaiol	Terpene	Leaves	Donati et al. [20]
Ledene	Terpene	Leaves	Donati et al. [20]
Linalol	Terpene	Leaves	Donati et al. [20]
Myrcene	Terpene	Leaves	Donati et al. [20]
Terpinen-4-ol	Terpene	Leaves	Donati et al. [20]
Terpineol	Terpene	Leaves	Donati et al. [20]
α-humulene (α-caryophyllene)	Terpene	Leaves	Donati et al. [20]
α-pinene	Terpene	Leaves	Donati et al. [20]
β-caryophyllene	Terpene	Leaves	Donati et al. [20]
β-element	Terpene	Leaves	Donati et al. [20]

**Table 4:** Antimicrobial activity *Schinopsis brasiliensis*.

Plant Part	Extract	Microorganism	MIC	Control	Reference
Barks	Hydroalcoholic	*E. faecalis*	0.25 mg/mL	Chlorhexidine	Sette-de-Souza et al. [23]
			0.5 mg/mL		
Barks	Ethanolic	*S. mutans*	0.5 mg/mL	Chlorhexidine	Sette-de-Souza et al. [24]
		*S. oralis*	0.5 mg/mL		
		*S. mitis*	0.5 mg/mL		
		*S. salivarius*	0.25 mg/mL		
Seeds	Ethanolic	*S. choleraesuis*	37.32 mg/mL	Tetracycline, Nystatin solution	Farias et al. [25]
Barks	Hydroalcoholic	*S. aureus*	50 mg/mL	Malachite Green dye	Formiga-Filho et al. [26]
		*Escherichia*	500 mg/mL		
		*P. aeruginosa*	50 mg/mL		
		*E. faecalis*	200 mg/mL		
Leaves	Hydroalcoholic	*S. aureus*	50 mg/mL	Malachite Green dye	Formiga-Filho et al. [26]
		*E. coli*	200 mg/mL		
		*P. aeruginosa*	50 mg/mL		
		*E. faecalis*	100 mg/mL		
Barks	Ethanolic	*B. cereus*	12.5 mg/mL	Gentamicin	Lima-Saraiva et al. [27]
		*E. coli*	12.5 mg/mL		
		*E. faecali*	12.5 mg/mL		
		*K. pneumoniae*	12.5 mg/mL		
		*P. aeruginosa*	12.5 mg/mL		
		*S. marcescens*	6.25 mg/mL		
		*S. flexneri*	3.12 mg/mL		
		*S. enterica*	0.39 mg/mL		
		*S. aureus*	3.12 mg/mL		
Leaves	Ethanolic	*S. haemolyticus*	0.17 mg/mL	Chloramphenicol, Erythromycin, Vancomycin, Oxacillin, Gentamicin, Tetracycline, Clindamycin, Penicillin	Ribeiro et al. [42]
		*S. aureus*	0.17 mg/mL		
		*E. coli*	0.17 mg/mL	Chloramphenicol, Ampicillin, Gentamicin, Ciprofloxacin, Tetracycline, Norfloxacin	
Leaves	Hydroalcoholic	*E. coli*	0.23 µg/mL	Ceftriaxone	Oliveira et al. [43]
		*K. pneumoniae*	10 µg/mL		
Leaves, Flowers, Root, Bark, Fruits	Methanolic	*S. aureus*	0.125 mg/mL	Tetraciclin	Saraiva et al. [33]
	Ethyl Acetate		0.25 mg/mL		
Leaves	Methanolic	*E. coli*	250 µg/mL	Tetracycline, Gentamycin, Ketoconazole	Saraiva et al. [22]
		*E. faecalis*	2 µg/mL		
		*S. aureus*	125 µg/mL		
		*S. saprophyticus*	500 µg/mL		
		*S. epidermidis*	500 µg/mL		
		*P. aeruginosa*	31.25 µg/mL		
Leaves	Ethyl Acetate	*S. aureus*	100 µg/mL	Tetracycline, Oxacilin	Saraiva et al. [6]
		*E. coli*	>100 µg/mL		
		*K. pneumoniae*	>100 µg/mL		
		*E. faecalis*	>100 µg/mL		
		*Salmonella spp*	>100 µg/mL		
Leaves	Methanolic	*S. aureus*	25 µg/mL		Saraiva et al. [6]
		*E. coli*	50 µg/mL		
		*K. pneumoniae*	100 µg/mL		
		*E. faecalis*	>100 µg/mL		
		*Salmonella spp*	>100 µg/mL		
		*C. albicans*	200 µg/mL	Ketoconazole	
		*C. krusei*	200 µg/mL		
		*C. tropicalis*	200 µg/mL		
Barks	Hydroalcoholic	*P. aeruginosa*	0.004 µL/µL	Chlorhexidine	Silva et al. [1]
		*E. faecalis*	1 µL/µL		
		*S. aureus*	0.063 µL/µL		
		*S. oralis*	0.5 µL/µL		
Leaves	Ethanolic	*S. aureus*	1.04 mg/mL	Erythromycin	Silva et al. [44]
Barks	Ethanolic	*S. aureus*	1.04 mg/mL	Erythromycin	Silva et al. [44]
Root bark	Hexane	*S. aureus*	>1000 µg/mL	-	Moreira et al. [19]
Root bark	Chloroform	*S. aureus*	31.25 µg/mL	-	Moreira et al. [19]
Root bark	Ethyl Acetate	*S. aureus*	62.50 µg/mL	-	Moreira et al. [19]
Root bark	Butanol	*S. aureus*	125 µg/mL	-	Moreira et al. [19]
Heartwood	Hexane	*S. aureus*	>1000 µg/mL	-	Moreira et al. [19]
Heartwood	Chloroform	*S. aureus*	250 µg/mL	-	Moreira et al. [19]
Heartwood	Ethyl Acetate	*S. aureus*	62.50 µg/mL	-	Moreira et al. [19]
Heartwood	Butanol	*S. aureus*	250 µg/mL	-	Moreira et al. [19]

**Table 5:** Antioxidant activity of *Schinopsis brasiliensis*.

Plant Part	Extract	Method	Main Result	Reference
Bark	Ethanolic	DPPH	IC_50_: 1.46 ± 0.07 µg/mL	Lima-Saraiva et al. [27]
Bark	Ethanolic	β-carotene	60.81%	Lima-Saraiva et al. [27]
Bark	Ethanolic	TEAC	3.04 mg/mL	Santos et al. [21]
Bark	Ethanolic	DPPH	IC50: 19.69 ± 0.77 µg/mL	Almeida-Andrade et al. [28]
Leaf	Essential Oil	ORAC	1918, 3 ± 246 µmol/g	Donati et al. [20]
Leaf	Essential Oil	DPPH	IC_50_: 17.63 mg/mL (9.19–33.82)	Donati et al. [20]
Leaf	Methanolic	DPPH	EC50 = 8.80 ± 0.94 g/mL	Saraiva et al. [22]
Root bark	Hexane	DPPH	>1000 µg/mL	Moreira et al. [19]
Root bark	Chloroform	DPPH	101.53 µg/mL	Moreira et al. [19]
Root bark	Ethyl Acetate	DPPH	38.37 µg/mL	Moreira et al. [19]
Root bark	Butanol	DPPH	53.46 µg/mL	Moreira et al. [19]
Root bark	Hexane	β-carotene	39.64 µg/mL	Moreira et al. [19]
Root bark	Chloroform	β-carotene	115.74 µg/mL	Moreira et al. [19]
Root bark	Ethyl Acetate	β-carotene	127.16 µg/mL	Moreira et al. [19]
Root bark	Butanol	β-carotene	29.65 µg/mL	Moreira et al. [19]
Heartwood	Hexane	DPPH	>1000 µg/mL	Moreira et al. [19]
Heartwood	Chloroform	DPPH	85.54 µg/mL	Moreira et al. [19]
Heartwood	Ethyl Acetate	DPPH	36.49 µg/mL	Moreira et al. [19]
Heartwood	Butanol	DPPH	71.43 µg/mL	Moreira et al. [19]
Heartwood	Hexane	β-carotene	301.51 µg/mL	Moreira et al. [19]
Heartwood	Chloroform	β-carotene	190.81 µg/mL	Moreira et al. [19]
Heartwood	Ethyl Acetate	β-carotene	31.42 µg/mL	Moreira et al. [19]
Heartwood	Butanol	β-carotene	109.72 µg/mL	Moreira et al. [19]

DPPH: 2,2-Diphenyl-1-Picryl-Hydrazyl; TEAC: Trolox Equivalent Antioxidant Capacity; ORAC: Oxygen Radical Absorbance Capacity; IC_50_: Inhibitory Concentration; EC_50_: Efficient Concentration.

**Table 6:** Toxicity tests of *S. brasiliensis* extract against different experimental models.

Study Desing	Plant Part	Extract	Experimental Models	LC_50_/IC50	Reference
In vivo	Bark	Ethanolic	*Artemia salina*	LC_50_ > 100 μg/mL	Santos et al. [46]
In vivo	Bark	Methanolic	*Artemia salina*	LC_50_ > 100 μg/mL	Santos et al. [46]
In vivo	Bark	Chloroform	*Artemia salina*	LC_50_ = 313 μg/mL	Santos et al. [46]
In vivo	Bark	Hexane	*Artemia salina*	LC_50_ = 582 μg/mL	Santos et al. [46]
In vivo	Bark	Ethyl acetate	*Artemia salina*	LC_50_ = 557 μg/mL	Santos et al. [46]
In vivo	Bark	Hydroalcoholic	*Artemia salina*	LC_50_: 428 µg/mL	Silva et al. [1]
In vivo	Leaf	Methanolic	*Artemia salina*	LC_50_: 705.54 ± 60.46 μg/mL	Saraiva et al. [22]
In vivo	Leaf	Ethanolic	*Artemia salina*	LC_50_: 512 μg/mL	Silva et al. [44]
In vivo	Seed	SPF	*Ceriodaphnia dubia*	LC_50_: 1.91 mg/mL	Barbosa et al. [47]
In vivo	Seed	Ethanolic	*Artemia sp*	LC_50_: 962.97 μg/mL	Souza et al. [45]
In vitro	Seed	SPF	Fibroblasts 3T3	LC_50_: 6.14 mg/mL	Barbosa et al. [47]
In vitro	Leaf	Hydroalcoholic	Glioblastoma SF-295	IC_50_ = 78.57 μg/mL	Reis-Luz et al. [39]
In vitro	Leaf	Hydroalcoholic	Prostate PC3	IC_50_ = 71.54 μg/mL	Reis-Luz et al. [39]
In vitro	Leaf	Hydroalcoholic	Leukemia HL60	IC_50_ = 52.58 μg/mL	Reis-Luz et al. [39]
In vitro	Leaf	Hydroalcoholic	Colorectal RAJI	IC_50_ = 55.90 μg/mL	Reis-Luz et al. [39]
In vitro	Leaf	Hydroalcoholic	Colorectal HCT-116	IC_50_ = 61.73 μg/mL	Reis-Luz et al. [39]
In vitro	Leaf	Hydroalcoholic	Colorectal SW-620	IC_50_ = 65.46 μg/mL	Reis-Luz et al. [39]
In vitro	Leaf	Hydroalcoholic	Fibroblast L929	IC_50_ = 49.53 μg/mL	Reis-Luz et al. [39]
In vitro	Bark	Hydroalcoholic	Glioblastoma SF-295	IC_50_ > 100 μg/mL	Reis-Luz et al. [39]
In vitro	Bark	Hydroalcoholic	Prostate PC3	IC_50_ > 100 μg/mL	Reis-Luz et al. [39]
In vitro	Bark	Hydroalcoholic	Leukemia HL60	IC_50_ = 58.75 μg/mL	Reis-Luz et al. [39]
In vitro	Bark	Hydroalcoholic	Colorectal RAJI	IC_50_ > 100 μg/mL	Reis-Luz et al. [39]
In vitro	Bark	Hydroalcoholic	Colorectal HCT-116	IC_50_ = 93.64 μg/mL	Reis-Luz et al. [39]
In vitro	Bark	Hydroalcoholic	Colorectal SW-620	IC_50_ = 25.68 μg/mL	Reis-Luz et al. [39]
In vitro	Bark	Hydroalcoholic	Fibroblast L929	IC_50_ = 82.00 μg/mL	Reis-Luz et al. [39]

SPF = Sodium phosphate buffer.

**Table 7:** Other biological activity from *Schinopsis brasiliensis*.

Biological Activity	Plant Part	Extract	Method (Study Design)	Main Results	IC50	Reference
Photoprotection	Bark	Ethanolic	Espectrophotometric (in vitro)	SPF: 6.26 ± 0.28	-	Almeida-Andrade et al. [28]
	Bark	Ethanolic	SPF (in vitro)	SPF: 6 UVB	-	Lima-Saraiva et al. [27]
Preserving agent	Leaf	Hydroalcoholic	DSC and FT-IR (in vitro)	-	-	Fernandes et al. [48]
Molluscicide (*Biomphalaria glabrata*)	Bark	Chloroform Ethyl Acetate	Santos and Sant’Ana (2001) (in vivo)	LC_90_: 68 μg/mL	-	Santos et al. [46]
				LC_90_: 73 μg/mL		
Larvicidal (*Aedes aegypti*)	Bark	Ethyl Acetate Hexane Chloroform	WHO (in vivo)	LC_50_: 345 μg/mL LC_50_: 527 μg/mL LC_50_: 583 μg/mL	-	Santos et al. [46]
	Seed	Ethanolic	WHO (in vivo)	FC strain: 100% SS strain: 100%	FC strain: 580.9 µg/mL SS strain: 661.6 µg/mL	Souza et al. [45]
	Seed	Sodium phosphate buffer	Konishi et al., 2008 and WHO adapted (in vivo)	100% of dead	-	Barbosa et al. [47]
Pupicidal (*Aedes aegypti*)	Seed	Ethanolic	WHO (in vivo)	FC strain: 100% SS strain: 100%	FC strain: 32.9 µg/mL SS strain: 40.6 µg/mL	Souza et al. [45]
	Seed	Sodium phosphate buffer	Konishi et al., 2008 and WHO adapted (in vivo)	100% of dead	-	Barbosa et al. [47]
Ovicidal (*Aedes aegypti*)	Seed	Ethanolic	WHO (in vivo)	FC strain: 5.7% SS strain: 0%	-	Souza et al. [45]
	Seed	Sodium phosphate buffer	Konishi et al., 2008 and WHO adapted (in vivo)	ODI_2.5%_ 25.44 ODI_20%_ 51.10	-	Barbosa et al. [47]
Anti-inflammatory	Bark	Hydroethanolic	Carrageenan (in vivo)	EAF: 100 mg/kg Agal: 10 mg/kg	-	Santos et al. [18]
	Root Bark	Methanolic	Carrageenan (in vivo)	-	-	Moreira et al. [19]
	Heartwood	Methanolic	Carrageenan (in vivo)	-	-	Moreira et al. [19]
Antinociceptive	Bark	Hydroethanolic	Formalin-induced licking (in vivo)	EAF: 40% less pain. HEE: 40% less pain	-	Santos et al. [18]
	Root Bark	Methanolic	Formalin-induced and paw edema (in vivo)	-	-	Moreira et al. [19]
	Heartwood	Methanolic	Formalin-induced and paw edema (in vivo)	-	-	Moreira et al. [19]
Anti-hemolytic	Bark	Ethanolic		43.84% ± 0.02	-	Lima-Saraiva et al. [27]
	Bark	Hydroalcoholic	Cruz-Silva et al., 2000 (in vitro)	-	92.66 mg/mL	Sette-de-Souza et al. [23]
	Bark	Ethanolic	Cruz-Silva et al., 2000 (in vitro)	-	50.27 mg/mL	Sette-de-Souza et al. [24]
Enzymatic inhibitor	Seed	Sodium phosphate buffer		Trypsin: 282.33	-	Barbosa et al. [47]
				Chymotrypsin: 90.42	-	
				Proteases: 141.17	-	
				Amylase: 26.50	-	

SPF: Sun Protection Factor; DSC: Differential Scanning Calorimetry; FT-IR: Fourier-transform infrared spectroscopy; UVB: Ultraviolet B radiation; LC: Lethal Concentration; FC: Field-collected; SS: susceptible to temephos; ODI: oviposition deterrence index; Agal: Chromatographic analysis of gallic acid; EAF: ethyl acetate fraction; HEE: hydroethanol extract.

## Data Availability

The data presented in this study are available in article.
